# Enhanced recovery after surgery (ERAS) programs for esophagectomy protocol for a systematic review and meta-analysis

**DOI:** 10.1097/MD.0000000000010016

**Published:** 2018-02-23

**Authors:** Feiyu Liu, Wei Wang, Chengde Wang, Xiaonu Peng

**Affiliations:** a Department of Pharmacy; b Department of Thoracic Surgery, The Affiliated Yantai Yuhuangding Hospital of Qingdao University, Yantai, Shandong, China; c Department of Thoracic and Cardiovascular Surgery, The University of Texas MD Anderson Cancer Center, Houston, TX, USA.

**Keywords:** enhanced recovery after surgery, ERAS, esophageal neoplasms, esophagectomy

## Abstract

**Background:**

Esophageal cancer is one of the worst malignant digestive neoplasms with poor treatment outcomes. Esophagectomy plays an important role and offers a potential curable chance to these patients. However, esophagectomy with radical lymphadenectomy is known as one of the most invasive digestive surgeries which are associated with high morbidity and mortality. The enhanced recovery after surgery (ERAS) protocol is a patient-centered, surgeon-led system combining anesthesia, nursing, nutrition, and psychology, which is designed for reducing complications, promoting recovery, and improving treatment outcomes. This systematic review and meta-analysis is aiming at how beneficial, and to what extent ERAS really will be.

**Methods:**

A systematic literature search will be performed through January 2018 using MEDLINE, EMBASE, the Cochrane Central Register of Controlled Trials, and Google Scholar for relevant articles published in any language. Randomized controlled trials, prospective cohort studies, and propensity-matched comparative studies will be included. All meta-analyses will be performed using Review Manager software. The quality of the studies will be evaluated using the guidelines listed in the Cochrane Handbook. The Preferred Reporting Items for Systematic Reviews and Meta-Analyses statements will be followed until the findings of the systematic review and meta-analysis are reported.

**Results:**

The results of this systematic review and meta-analysis will be published in a peer-reviewed journal.

**Conclusion:**

Our study will draw an objective conclusion of the comparisons between ERAS and conventional care in aspects of perioperative outcomes and provide level I evidences for clinical decision makings.

## 1 Introduction

Esophageal cancer is the ninth most commonly diagnosed cancer and the sixth most common cause of cancer-related deaths worldwide in 2013.^[1]^ And it is one of the worst malignant digestive neoplasms with poor treatment outcomes. Esophagectomy is the mainstay of curative treatment strategies for localized esophageal cancer,^[2–5]^ which plays an important role and offers a potential curable chance to these patients. However, esophagectomy with radical lymphadenectomy is known as one of the most invasive digestive surgeries which are associated with high morbidity and mortality.^[6,7]^ Therefore, ideas to reduce complications, promote recovery, and improve treatment outcomes seem to be very attractive.

The enhanced recovery after surgery (ERAS) program, also known as fast track surgery (FTS), is a patient-centered, surgeon-led system combining anesthesia, nursing, nutrition and psychology, which was initiated by Henrik Kehlet in the 1990s.^[8,9]^ It aims to minimize surgical stress, reduce surgery-related complications, and accelerate postoperative recovery during the perioperative period. The ERAS program has been successfully implemented in various surgically treated diseases, especially in colorectal surgeries.^[10–12]^ However, ERAS developed relatively late in esophagectomy due to its surgical complexity and high morbidity of postoperative complications. Recently, with the popularization of minimally invasive esophagectomy, attention to organ function protection concepts and improvement of gastric conduit and anastomosis techniques, ERAS has gradually developed in the field of esophagectomy.^[13–16]^

Two systematic reviews with meta-analysis described the feasibility and safety of ERAS in patients undergoing esophagectomy compared with conventional care.^[17,18]^ Both of them involved only 1 randomized controlled trial (RCT), and most of the studies analyzed were of low quality with high risks of bias, so the level of evidences was limited. Pisarska et al^[17]^ reported there were significant differences in nonsurgical complications and pulmonary complications between EARS group and conventional care. However, Markar et al^[18]^ got contradictory results in these aspects. It is still unclear how beneficial, and to what extent ERAS really is for esophagectomy. Due to some high quality studies were published recently, we can take this advantage to conduct a systematic review and meta-analysis with higher level of evidences.^[19–21]^ Moreover, in order to minimize the heterogeneity and bias, we will select RCTs and propensity-matched comparative studies which match across a range of baseline factors to generate 2 similar groups for comparison.

## 2 Objective

A systematic review and meta-analysis will be performed to assess the effects of ERAS protocol versus conventional care for patients undergoing esophagectomy.

## 3 Methods

This protocol for systematic review and meta-analysis is performed according to the Preferred Reporting Items for Systematic Review and Meta-Analysis Protocols (PRISMA-P) statement.^[22]^ This protocol has been registered in the PROSPERO network (registration number: CRD42018085977). The systematic review and meta-analysis will be reported according to the Preferred Reporting Items for Systematic Reviews and Meta-Analyses (PRISMA) guidelines.^[23]^

### 3.1 Eligibility criteria

#### 3.1.1 Types of participants

The included participants will be adults who were diagnosed with esophageal cancer histologically or cytologically confirmed and treated with esophagectomy. Comparisons of ERAS or FTS with the conventional care were evaluated. There will be no restrictions regarding sex, race/ethnicity, education and economic status, and no restriction in publication language.

#### 3.1.2 Types of studies

We propose to include studies that report comparisons between ERAS and non-ERAS treatment for patients undergoing esophagectomy. RCTs, prospective cohort studies, and propensity-matched comparative studies will be used for the qualitative and quantitative synthesis of the systematic review.

#### 3.1.3 Exclusion criteria

Non-peer-reviewed articles, review articles, case reports, case series, animal studies, meeting abstracts, letters to the editor, commentaries, editorials, proceedings, non-propensity-matched comparative studies, and other nonrelevant studies will be excluded from analysis.

### 3.2 Information sources

We will perform a systematic literature search through January 2018 using MEDLINE, EMBASE, the Cochrane Central Register of Controlled Trials, and Google Scholar for relevant articles published in any language.

### 3.3 Search strategy

The relevant searching terms will match Medical Subject Heading terms, and the searches will be repeated immediately before the final analyses to identify additional studies for inclusion. An example of the PubMed search strategy is shown in Table 1.

**Table 1:**
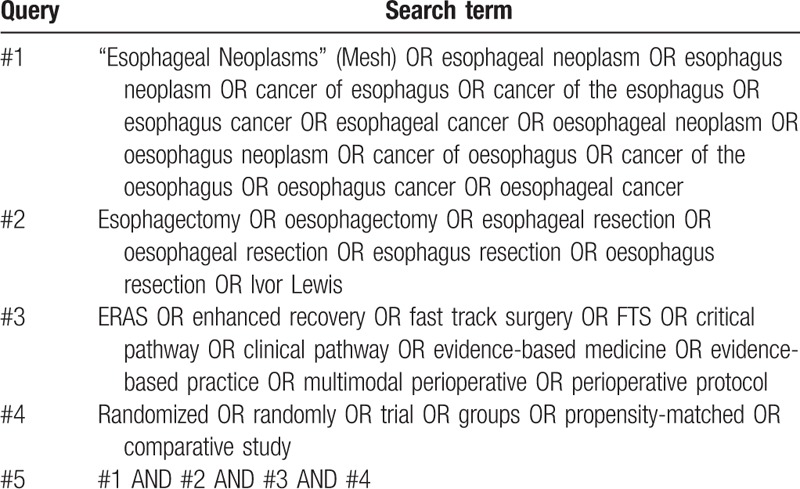
Search strategy for PubMed.

### 3.4 Study records

#### 3.4.1 Selection of studies

Two review authors (FL and WW) will independently screen titles and abstracts of all the potential studies to assess whether they meet the inclusion criteria as defined by the protocol. We will retrieve the full text of all potentially eligible studies and 2 review authors (FL and WW) will independently screen the full-text and identify studies for inclusion, and record reasons for exclusion of the ineligible studies. Any disagreement will be resolved through discussion or, if required, consultation with a third review author (CW or XP). Duplicates will be excluded and multiple reports of the same study will be integrated into 1 unit of interest in the review. The selection process will be recorded in sufficient detail to complete a PRISMA flow diagram and “Characteristics of excluded studies” table.^[24]^ We will not impose any language restrictions.

#### 3.4.2 Data extraction and management

Data will be extracted from the included studies by 3 authors (FL, WW, and CW) independently and recorded on a predesigned data collection form. We will extract the following study characteristics:

(1) *Study characteristics*: study design, number of study centers and locations, study setting, withdrawals, total duration of the trial, periods of data collection, follow-up duration, blanking periods.
(2) *Population characteristics*: inclusion and exclusion criteria, number, mean age, age range, gender, diagnostic criteria, pathological confirmation, staging of the tumor according to the AJCC TNM classification for esophageal cancer, type of surgical procedure.
(3) *Intervention characteristics*: relevant factors of preadmission counseling, preoperative preparation, anesthetic protocol and postoperative care.
(4) *Outcomes*: primary and secondary outcomes specified and collected, and time points reported.

### 3.5 Outcomes

#### 3.5.1 Primary outcome

The primary outcome measure of our systematic review is overall morbidity of complications.

#### 3.5.2 Secondary outcomes

The secondary outcomes are intraoperative blood loss, operation time, number of retrieved lymph nodes, postoperative hospital stay, specific complications rate, 30-day mortality, and readmission rate. The specific complications are as follows: pulmonary complications, cardiovascular complications, gastrointestinal complications, surgical technology related complication (anastomotic leakage).

### 3.6 Assessment of risk of bias

Three review authors (FL, WW, and CW) will independently assess the risk of bias for each study using the criteria outlined in the Cochrane Handbook for Systematic Reviews of Interventions. Any disagreements will be resolved by discussion or by involving another review author (XP). The risk of bias will be assessed according to the following domains: random sequence generation; allocation concealment; blinding of participants and personnel; blinding of outcome assessment; incomplete outcome data; selective outcome reporting; and other bias. Each potential source of bias will be graded as high, low or unclear and a quote from the study report with a justification for our judgement will be provided in the “Risk of bias” table. The risk of bias judgements across different studies for each of the domains listed will be summarized.

### 3.7 Data synthesis

Data from studies judged to be clinically homogeneous will be pooled using Review Manager 5.3 software. Heterogeneity between studies will be assessed using the Cochran's Q and Higgins *Ι*^2^ statistic. *P* < .10 for the Chi^2^ statistic or an *I*^2^ > 50% will be considered as showing considerable heterogeneity, and the data will be analyzed using the random-effect model. Otherwise, the fixed-effect model will be used. The Mantel–Haenszel method will be applied for pooling of dichotomous data and results will be presented as relative risk (RR) with their 95% confidence intervals (CI). Inverse variance method will be used for pooling of continuous data and results will be presented as standardized mean difference (SMD) with their 95% CI. A *P* < .05 will be considered significant.

#### 3.7.1 Subgroup analysis

If data are sufficient, we will conduct subgroup analyses between different surgical procedures: open surgery and minimally invasive surgery. Subgroup analyses will also be performed to explore potential sources of heterogeneity.

#### 3.7.2 Sensitivity analysis

A sensitivity analysis will be performed to confirm whether the pooled results are robust and credible by excluding highly biased studies.

#### 3.7.3 Dealing with missing data

In the condition of missing or unclear data, study authors will be contacted at the eligibility assessment and/or data extraction stage. Secondary publications may be considered as missing data if they have the same study population.

### 3.8 Publication bias

Egger's regression test will be performed to assess the publication bias of the included studies.^[25]^ If there is a publication bias, trim and fill analysis will be performed.

### 3.9 Evidence eveluation

The evidence grade will be determined by using the guidelines of the GRADE (Grading of Recommendations, Assessment, Development, and Evaluation) system using 4 levels—high quality, moderate quality, low quality, and very low quality.^[26]^

## 4 Discussion

This protocol presents the methodology of a systematic review for assessing the feasibility and safety of ERAS programs for patients undergoing esophagectomy. We will comprehensively search, screen, assess, and extract valuable data from several databases as previously mentioned, and report this review results according to the PRISMA guidelines.

To our knowledge, this will be the first systematic review and meta-analysis using data of randomized controlled trials and propensity-matched comparative studies to compare the clinical outcomes between ERAS and conventional care for patients undergoing esophagectomy updating to January 2018. The aim of our study is to draw an objective conclusion of the comparisons in aspects of perioperative outcomes and provide physicians level I evidences for clinical decision makings.

## 5 Contributors

FL, WW, and XP conceived and designed this study. FL and WW drafted the protocol. FL, WW, and CW will conduct the search, data screening and extraction. FL, WW, CW, and XP have critically reviewed the manuscript and approved it for publication.
